# Association between risk perception of complications and self-efficacy among young and middle-aged patients with type 2 diabetes in China: a cross-sectional study

**DOI:** 10.3389/fpsyg.2026.1711439

**Published:** 2026-01-22

**Authors:** Gang Chen, Yuhan Wang, Youyou Zhai, Xin Kong, Yuying Wang

**Affiliations:** 1 Department of Ophthalmology, The First Affiliated Hospital of Zhengzhou University, Zhengzhou, China; 2 Department of Cardiovascular Surgery, West China Hospital, Sichuan University, Chengdu, China

**Keywords:** perception, psychological, risk, self-efficacy, type 2 diabetes mellitus, young and middle-aged

## Abstract

**Background:**

Risk perception refers to an individual’s subjective judgment and evaluation of the characteristics and severity of risk-related matters, serving as a crucial predictive factor influencing personal health behaviors and self-management capabilities. Therefore, accurate health risk perception is essential for improving preventive health behaviors and reducing the occurrence of complications in diabetic patients. This study aimed to delve into the current status and factors influencing risk perception of diabetic complications among young and middle-aged patients with type 2 diabetes mellitus (T2DM).

**Methods:**

A descriptive cross-sectional survey was adopted to select young and middle-aged patients with T2DM who were admitted to two tertiary hospitals in Zhengzhou, Henan, China, from February 2024 to October 2024. General information questionnaire, risk perception survey-diabetes mellitus (RPS-DM), and Diabetes Self-Efficacy Scale (DSES) were employed for data collection. Data were analyzed using descriptive statistics, *t*-tests, one-way analysis of variance (ANOVA), Pearson correlation analysis, and multiple linear regression.

**Results:**

A total of 420 valid responses were received. The score of the risk perception survey-diabetes mellitus was 52.32 ± 10.76, and the score of self-efficacy was 22.73 ± 4.49. Pearson correlation analysis revealed a statistically significant positive correlation (*r* = 0.53, *p* < 0.001) between risk perception and self-efficacy among young and middle-aged patients with T2DM. Moreover, the multiple linear regression analysis indicated that education level, family history of diabetes, and number of comorbid chronic diseases were the influencing factors of risk perception of diabetic complications (all *p* < 0.05).

**Conclusion:**

The risk perception level was positively correlated with self-efficacy of diabetes. Patients of lower educational levels, without a family history of diabetes, more than three comorbidities, and lower self-efficacy are indicative of lower levels of risk perception. Identifying these influencing factors can provide references and support for developing individualized support and intervention measures to enhance their awareness and perception of complication risks.

## 1 Introduction

In 2021, the International Diabetes Federation (IDF) reported that over 140 million people in China had diabetes, with T2DM accounting for over 90% of cases and ranking first globally. Of these patients, only 49.2% had received professional treatment (Li et al., 2020; Sun et al., 2022). The onset of diabetes mellitus has been trending toward younger ages in recent years, and the fastest-growing demographic in terms of prevalence rates is young and middle-aged individuals (Yang et al., 2024). People under 60 account for nearly half of all diabetes-related deaths worldwide each year (Duncan et al., 2025). According to data, about 6.7 million people in China lost their lives to diabetes or its complications in 2021, making diabetic complications the leading cause of death among diabetic patients (Guo, 2022). Since there are frequently no obvious symptoms in the early stages of diabetic complications, patients may underestimate their risks, which could ultimately lead to complications. Therefore, for young and middle-aged patients with T2DM, early detection and proactive management of diabetic complications are essential (Zhang et al., 2017).

Risk perception is a key predictor that influences people’s health behaviors and capacity for self-management. In the medical field, it is defined as people’s subjective assessment and judgment of the traits and seriousness of risk factors. According to research, diabetic patients’ self-management skills can be improved by adopting better disease management and preventive health practices when they have a realistic understanding of their health risks (Nie et al., 2018; Riedinger et al., 2022). On the other hand, patients who do not have a realistic understanding of the risks associated with their disease are likely to make irrational medical decisions, miss out on the best possible treatment, and see their condition worsen as a result (Yang et al., 2021). According to studies, Chinese patients with T2DM who are young or middle-aged typically do not know the risk factors that can result in complications, and they perceive fewer risks of complications than people in other age groups (Wang et al., 2021; Li et al., 2023). Without a doubt, this hastens the development and course of diabetic complications.

In the field of health behavior, self-efficacy can be defined as a person’s belief in their ability to successfully perform certain health-related behaviors in various situations, which affects the behavioral choices, level of effort, and persistence (McAnally and Hagger, 2023). Studies conducted previously among patients with AIDS (Yuan et al., 2025), coronary artery diseases (Kang and Yang, 2013), stroke (Zhang et al., 2025), and breast cancer (Ziner et al., 2012) have indicated that high self-efficacy serves as a protective factor in risk perception, with patients exhibiting higher levels of self-efficacy demonstrating elevated perception of risks. However, few studies have investigated the relationship between the perception of complication risks and self-efficacy in young and middle-aged patients with T2DM. Therefore, this study aims to delve into the current status of risk perception of diabetic complications among this population and explore the effects of demographic and self-efficacy on risk perception of diabetic complications in young and middle-aged patients with T2DM. Further enhance patients’ perception of complication risks and provide a basis for healthcare professionals to develop personalized intervention strategies for preventing the occurrence of diabetic complications.

## 2 Methods

### 2.1 Study design and settings

This study used a cross-sectional design and a convenience sampling method to select young and middle-aged patients with T2DM who were admitted to two tertiary hospitals in Henan Province from February 2024 to October 2024. The collection of questionnaires was conducted by investigators who had received uniform training, including a general information questionnaire, risk perception survey-diabetes mellitus (RPS-DM), and Diabetes Self-Efficacy Scale (DSES). Before the survey, participants were briefed about the purpose, significance, questionnaire completion process, and instructions of the study, and they signed informed consent forms. The eligibility criteria for participants include: (1) meet the diagnostic and categorization criteria of Guideline for the prevention and treatment of diabetes mellitus in China (2024 edition) (Chinese Diabetes Society, 2025); (2) 18 ~ <60 years of age; (3) conscious, normal reading and comprehension ability, and informed consent for the study. Exclusion criteria include: (1) the patient has been diagnosed with severe organ failure; (2) a history of mental illness or psychological disorders; (3) participation in other studies.

### 2.2 Participants and procedure

In estimating the sample size, the primary reference is the sample estimation method of Castro and Kendall, which ensures that the sample size is at least 10–15 times the number of variables. There were 14 independent variables in this study. Considering a 20% sample loss rate, the minimum sample size was [14 × 10÷(1%–20%)] = 175. A total of 420 young and middle-aged patients with T2DM participated. All participants provided informed consent and agreed to take part in the study.

### 2.3 Measurement

#### 2.3.1 Sociodemographic data and clinical data

The demographic and clinical characteristics questionnaire developed by the study team was used to obtain demographic data (gender, age, education level, residence, marital status) and disease-related data (family history of diabetes, duration of diabetes, BMI, treatment methods, and number of comorbid chronic diseases). The demographic data were filled in by the patients, and disease-related data were exported from the hospital’s electronic medical record system.

#### 2.3.2 Risk perception survey-diabetes mellitus (RPS-DM)

The risk perception survey-diabetes mellitus (RPS-DM), developed by Walker et al. (2007), is a simple scale that assesses the level of perception of patients with T2DM about the occurrence of diabetic complications. The Chinese version of the RPS-DM is a 23-item self-report scale (Ma et al., 2022), including five dimensions: personal control, worry, optimistic bias, personal risk of disease, and comparative environmental risk. Each item is rated on a 4-point Likert scale (from 1 = strongly disagree to 4 = strongly agree). Scores were calculated for each dimension, with higher scores suggesting higher levels of risk perception. In this study, the Cronbach’s *α* for this scale was 0.918.

#### 2.3.3 Diabetes self-efficacy scale (DSES)

The diabetes self-efficacy scale was developed by Ritter et al. (2016), and for this study, we employed the Chinese version of the scale translated by Wei (2013), is a simple scale that assesses the self-efficacy level of diabetic patients. It is a 9-item self-report scale, including four dimensions: diet, exercise, blood glucose management, and disease control. Each item is rated on a 5-point Likert scale (from 1 = completely lack confidence to 5 = completely confident). Scores were calculated for each dimension, with higher scores suggesting higher levels of self-efficacy. In this study, the Cronbach’s *α* for this scale was 0.873.

### 2.4 Data analysis

The collected data were entered by two individuals using Excel and analyzed with SPSS 26.0 statistical software. Categorical data were described using frequencies and percentages (%), while normally distributed continuous data were presented as the mean with standard deviation (*x* ± SD). The scores of risk perception among young and middle-aged patients with T2DM with different demographic characteristics were compared using independent-sample *t*-tests or one-way analysis of variance (ANOVA). Pearson correlation analysis was performed to determine the correlations between risk perception and self-efficacy. A *p*-value < 0.05 indicated a statistically significant difference.

## 3 Results

### 3.1 Participants’ characteristics

In this study, a total of 435 questionnaires were distributed. Among them, 15 questionnaires had incomplete responses and were eliminated. Eventually, a total of 420 valid questionnaires were recovered, with a valid recovery rate of 96%. Mean age of the patients was 48.56 ± 8.43 years, a total of 52.38% were male, 29.29% were under 45 years old, 48.57% with education level of junior high school and below, 55.24% resided in rural areas, 83.1% were married, 67.62% had duration of diabetes above 5 years, 39.52% had family history of diabetes, 53.57% of the patients had a BMI > 24, 47.62% were oral antidiabetic drugs and insulin, 51.19% had number of comorbid chronic diseases above 3. More comprehensive demographic and disease characteristics of patients can be found in Table 1.

**Table 1:** Distribution of patients according to sociodemographic and clinical characteristics.

Variable	Group	Number (n)	Percentage (%)
Gender	Male	220	52.38
	Female	200	47.62
Age (years)	<45	123	29.29
	45–59	297	70.71
Education level	Junior high school and below	204	48.57
	High school or junior college	146	34.76
	College and above	70	16.67
Residence	Rural	232	55.24
	Urban	188	44.76
Marital status	Unmarried	19	4.52
	Married	349	83.1
	Divorced	35	8.33
	Widowed	17	4.05
Duration of diabetes (years)	<5	136	32.38
	5–10	174	41.43
	>10	110	26.19
Family history of diabetes	Yes	166	39.52
	No	254	60.48
BMI (kg/m^2^)	<18.5	33	7.86
	18.5–23.9	162	38.57
	24–27.9	169	40.24
	≥28	56	13.33
Treatment methods	Oral antidiabetic drugs	62	14.76
	Insulin	158	37.62
	Oral antidiabetic drugs and insulin	200	47.62
Number of comorbid chronic diseases	<3	205	48.81
	3–5	146	34.76
	>5	69	16.43

### 3.2 The scores and univariate analysis of risk perception in young and middle-aged patients with T2DM

Among 420 young and middle-aged patients with T2DM, the mean risk perception score was 52.32 ± 10.76, and the average score of entries was 2.27 ± 0.47. As indicated in Table 2, the findings of the univariate analysis revealed that gender, education level, residence, family history of diabetes, and number of comorbid chronic diseases had significantly different scores of risk perception (*p* < 0.05).

**Table 2:** T/ANOVA tests of risk perception and participant characteristics.

Variable	Group	Mean (SD)	F/t	*P*
Gender	Male	51.03 (10.13)	−2.609	0.009
	Female	53.75 (11.26)		
Age (years)	<45	50.37 (10.27)	0.859	0.391
	45–59	53.13 (10.87)		
Education level	Junior high school and below	47.27 (8.87)	65.226	<0.001
	High school or junior college	55.32 (8.08)		
	College and above	60.80 (12.91)		
Residence	Urban	51.38 (11.32)	−2.043	0.042
	Rural	53.49 (9.92)		
Marital status	Unmarried	54.63 (12.95)	2.534	0.056
	Married	52.44 (10.90)		
	Divorced	53.09(7.83)		
	Widowed	45.71 (8.48)		
Duration of diabetes (years)	<5	50.69 (12.92)	2.808	0.061
	5–10	52.61 (9.69)		
	>10	53.88 (9.12)		
Family history of diabetes	Yes	48.68 (11.10)	−5.684	<0.001
	No	54.70 (9.85)		
BMI (kg/m^2^)	<18.5	48.73 (14.14)	1.776	0.151
	18.5–23.9	52.02 (10.69)		
	24–27.9	53.33 (9.99)		
	≥28	52.27 (10.72)		
treatment methods	Oral antidiabetic drugs	49.56 (10.54)	2.484	0.085
	Insulin	52.56 (9.83)		
	Oral antidiabetic drugs and insulin	52.60 (10.42)		
Number of comorbid chronic diseases	<3	49.15 (9.72)	28.402	<0.001
	3–5	53.37 (9.86)		
	>5	59.54 (11.69)		

### 3.3 Correlation analysis of risk perception and self-efficacy in young and middle-aged patients with T2DM

The score of self-efficacy was 22.73 ± 4.49. Based on the findings of this study (Table 3), correlation analysis between risk perception and self-efficacy showed that self-efficacy scores were positively correlated with the score of risk perception (*r* > 0.500, *p* < 0.01).

**Table 3:** Correlations analysis between risk perception and self-efficacy.

Variable	Risk perception (RPS-DM)	Personal control	Worry	Optimistic bias	Personal risk	Environmental risk
Diabetes self-efficacy scale (DSES)	0.530	0.431	0.338	0.398	0.499	0.355
Diet	0.486	0.379	0.307	0.361	0.461	0.331
Exercise	0.440	0.397	0.264	0.370	0.428	0.245
Blood glucose management	0.392	0.302	0.241	0.274	0.371	0.279
Disease control	0.471	0.380	0.326	0.338	0.421	0.338

### 3.4 Multiple linear regression analysis of risk perception in young and middle-aged patients with T2DM

Using the score of risk perception as the dependent variable, the five characteristic variables with statistical significance in the univariate analysis and the variables with statistical significance in the correlation analysis were taken as independent variables. Multiple linear regression analysis revealed that education level, family history of diabetes, number of comorbid chronic diseases, and the score of self-efficacy were the factors influencing the risk perception of young and middle-aged patients with T2DM (*p* < 0.05). Refer to Table 4 for details.

**Table 4:** Multiple linear regression analysis of factors influencing risk perception in young and middle-aged patients with T2DM.

Variable	B	SE	β	t	*P*	VIF
Constant	11.384	2.715		4.192	<0.001	
Gender	0.939	0.791	0.044	1.187	0.236	1.034
Education level	4.584	0.561	0.317	8.165	<0.001	1.149
Residence	1.265	0.801	0.059	1.579	0.115	1.051
Family history of diabetes	4.295	0.814	0.195	5.274	<0.001	1.05
Number of combined other diseases	2.212	0.555	0.152	3.989	<0.001	1.115
Diabetes self-efficacy scale (DSES)	0.854	0.094	0.356	9.057	<0.001	1.184

## 4 Discussion

To our knowledge, this study investigated the relationships between sociodemographic characteristics and self-efficacy and risk perception of complications from diabetes. The study found that individuals of higher education level, who have a family history of diabetes, a higher number of comorbid chronic diseases, and elevated levels of self-efficacy, have a higher level of risk perception of diabetic complications.

The results of this study indicated that the score of risk perception of diabetes complications in young and middle-aged patients with T2DM was 52.32 ± 10.76. There was an existence of optimistic bias for diabetes complications. Consistent with early studies (Calvin et al., 2011; Rouyard et al., 2017). The possible reasons for this could be that the study population consisted of young and middle-aged patients with T2DM. Previous research suggested that age serves as an independent risk factor for disease risk perception in diabetic patients (Nie et al., 2016). Older age is associated with a higher likelihood of developing multiple complications, thus prompting greater attention to personal health risks. On the other hand, young and middle-aged populations typically show comparatively greater physical functioning without significant discomfort or debilitation. In the early stages of diabetes, symptoms may be mild or even absent. This can lead patients to develop a psychological distance from diabetes-related complications, such as blindness, kidney failure, amputation, or heart disease, perceiving these complications as distant and fostering a lack of urgency to address them. Furthermore, young and middle-aged adults are in the midst of career advancement and facing the heaviest family responsibilities. The overwhelming pressures of daily life and work compel them to prioritize addressing immediate survival challenges, frequently pushing health management to a secondary position.

The study results indicated that an individual’s education level is a significant factor influencing risk perception among young and middle-aged patients with T2DM. Previous studies have shown that patients with higher levels of education exhibit a heightened awareness of their diabetes risk (Pelullo et al., 2019; Wang et al., 2024). One possible explanation for these findings could be that individuals with higher educational attainment have greater access to disease-related information channels. This may enable them to gain exposure to a broader range of healthcare options, develop stronger mastery of diabetes-related knowledge, and be more likely to understand both the main risk factors and protective elements regarding diabetes onset and its complications (Zhao, 2021).

The results of this study suggested that patients with T2DM with a family history of diabetes exhibit a higher risk perception of diabetic complications compared to those without a family history of diabetes. Consistent with earlier studies in other countries (Heidemann et al., 2019; Sulaiman et al., 2020). One possible explanation for these findings could be that patients with a family history of diabetes may directly experience and witness the harm of diabetes complications while caring for family members. They become more aware of their health problems as a result of this personal experience or observation, which leads them to prioritize preventive measures in their daily lives and maintain a higher level of vigilance against the risk of complications from diabetes.

The results of this study suggested that patients with T2DM with a greater number of comorbid chronic diseases exhibit a higher risk perception of diabetic complications. Aligning with the findings of Wang et al. (2024). In this study, 51.2% of diabetic patients had comorbidities of three or more chronic conditions; each additional comorbidity signifies increased complexity in disease management. The observed results may be attributed to the diabetic patients with multiple comorbidities (e.g., cardiovascular diseases, chronic kidney disease) will experience concurrent abnormalities across multiple organ systems (such as dyspnea in heart failure patients, edema in kidney disease patients, and limb numbness from neuropathy). These manifestations serve as persistent physiological warnings that continuously remind patients of their disease progression risk. Moreover, there exists a pathophysiological positive feedback loop between diabetes and other chronic diseases. Patients experienced the deteriorating interactions among multiple organ functions firsthand, leading to a heightened awareness of associated risks.

The findings of the study showed a positive correlation (*r* = 0.530, *p* < 0.001) between the perception of risk for complications from diabetes and diabetes self-efficacy. Patients with higher levels of self-efficacy were associated with a greater risk perception of diabetic complications, which is consistent with earlier studies (Chen, 2025). One possible explanation for these findings could be that diabetic patients with high self-efficacy generally demonstrate greater confidence and capability in learning, understanding, and managing diabetes-related knowledge, exhibiting stronger self-management skills and heightened risk awareness (Juarez et al., 2022). Furthermore, patients with high self-efficacy tend to attribute health outcomes, including the occurrence of complications, to factors within their control (such as diet, exercise, medication adherence, and health monitoring). They concurrently perceive complication risks as challenges to be actively managed rather than immutable threats.

This study has several limitations. The main limitation of our study was its cross-sectional nature; this cross-sectional study can merely analyze the factors related to risk perception, but not the causal relationship. Future studies with a longitudinal design are needed to assess the change trajectory of risk perception in young and middle-aged patients with T2DM over time, as well as the change of its associated factors. Furthermore, this study only discusses the beneficial factors arising from patients’ heightened awareness of complication risks. According to the risk compensation theory (Yang et al., 2018), the influence of high risk perception on patients may manifest dual effects. Overestimation of risk may lead individuals to be in a chronic stress state for a long time, causing adverse emotions such as mental depression and fear of disease. In the future, it is essential to focus on the psychological status of patients with high risk perception and investigate the relationship between their risk perception and negative emotions such as anxiety, depression, and fear of disease progression. In addition, we chose the convenience sampling method and recruited samples only from a central province in China, which may limit the representativeness of the samples. Future research should improve the sampling methods and expand the sample range to other provinces and cities to verify these results.

## 5 Conclusion

The current study underscores the need for improvement in risk perception of complications among young and middle-aged patients with T2DM, which shows a positive correlation with self-efficacy. Additionally, this perception is influenced by educational level, family history of diabetes, and the number of comorbid chronic diseases. Enhancing awareness of complication risks among young and middle-aged patients with T2DM requires collaborative efforts among patients, families, society, and healthcare institutions. It is recommended that healthcare professionals implement personalized health education tailored to individual characteristics during clinical practice. By enhancing patients’ sense of control (self-efficacy) over their disease, we aim to motivate them to proactively pay attention to the risk of complications and set small goals for patients to create successful experiences to build their confidence. Then, use tools such as risk calculators and peer demonstrations to concretize and personalize abstract risks, helping patients form a virtuous cycle from knowing to doing, thereby enhancing their health awareness and self-management skills to effectively prevent and delay the onset of diabetic complications.

## Data Availability

The original contributions presented in the study are included in the article/supplementary material, further inquiries can be directed to the corresponding authors.
